# 

**Published:** 1832-07-01

**Authors:** 


### CONTENTS

OF THE

### MEDICO-CHIRURGICAL REVIEW.

No. XXXIII. JULY 1, 1832.

### REVIEWS.

I.

A Treatise on the Injuries, the Diseases, and the Distortions of the Spine, &c. &c. By R. A. Stafford .. .. .. .. .. .. .. .. .. .. .. .. 1

II.

Lithotrity and Lithotomy compared—an Analytical Examination of the present Methods of treating Stone in the Bladder .. .. .. .. .. .. .. .. 24

II.—*(bis.)*

Observations on Surgery and Pathology. By W. J. Clement, Surgeon .. .. .. 43

III.

A Treatise on Retention of Urine, caused by Strictures in the Urethra, &c. By Theodore Ducamp; translated (with Notes) by W. H. Herbert, M.D. .. .. 49

IV.

Bombay Report on Epidemic Cholera .. .. .. .. .. .. .. .. .. .. 57

V.

Bengal Report of the Epidemic Cholera .. .. .. .. .. .. .. .. .. .. 72

VI.

A Manual of Midwifery. By Michael Ryan, M.D. .. .. .. .. .. .. .. 114

VII.

On the Epidemic Cholera of Newburn. By Dr. Craigie .. .. .. .. .. .. 121

VIII.

The Homoiopathic Doctrine .. .. .. .. .. .. .. .. .. .. .. .. 132

IX.

The Anatomy of the Thymus Gland. By Sir Astley Cooper, Bart. .. .. .. 139

### PERISCOPE.

1. Injection of Water into the Veins for Hydrophobia .. .. .. .. .. .. 145
2. On Arteritis. By Dr. Hope. With Commentaries .. .. .. .. .. .. 146
3. Messrs. Green and S. Cooper on Fracture of the Cervix Femoris .. .. .. 150
4. Singular Tumour from the Uterus—(Mr. Lawrence and Dr. Conquest.) .. .. 152
   *a.* Gonorrhœal Ophthalmia .. .. .. .. .. .. .. .. .. .. .. 153

*b.* Chimney Sweeper's Cancer, with Commentaries .. .. .. .. .. .. .. 153
*c.* Strangulated Hernia—Intestine wounded .. .. .. .. .. .. .. .. .. .. 156
5. M. Broussais on the Chronic Phlegmasiæ—(American Translation) .. .. 158
6. Dr. Kirk on Cholera Asphyxia .. .. .. .. .. .. .. .. .. .. .. .. .. 159

SPIRIT OF THE FOREIGN PERIODICALS.

7. Secred Remedies in France .. .. .. .. .. .. .. .. .. .. .. .. .. .. 161
8. Curious Fœtal Monstrosity .. .. .. .. .. .. .. .. .. .. .. .. .. .. 162
9. Gonorrhœa in Females .. .. .. .. .. .. .. .. .. .. .. .. .. .. .. 162
10. Poisoning by Arsenic.. .. .. .. .. .. .. .. .. .. .. .. .. .. .. .. 162
11. Electricity and Cholera .. .. .. .. .. .. .. .. .. .. .. .. .. .. .. 163
12. Auscultation in Midwifery .. .. .. .. .. .. .. .. .. .. .. .. .. .. 163
13. Actual Cautery, utility of.. .. .. .. .. .. .. .. .. .. .. .. .. .. .. 164
14. M. Dupuytren on Fracture of the Cervix Femoris .. .. .. .. .. .. .. 165
15. Chronic Ophthalmia .. .. .. .. .. .. .. .. .. .. .. .. .. .. .. .. 166
16. Sagacious Reflection .. .. .. .. .. .. .. .. .. .. .. .. .. .. .. .. 166
17. Baron Dupuytren's Letter on Cholera .. .. .. .. .. .. .. .. .. .. .. 166
18. Disease of Pylorus .. .. .. .. .. .. .. .. .. .. .. .. .. .. .. .. 168
19. Scrofula cured by Iodine .. .. .. .. .. .. .. .. .. .. .. .. .. .. .. 169
20. Natural History of Man .. .. .. .. .. .. .. .. .. .. .. .. .. .. .. 170
21. Abscess in the Heart .. .. .. .. .. .. .. .. .. .. .. .. .. .. .. 170
22. On the Decomposition of the Animal Structure .. .. .. .. .. .. .. .. 170
23. Dr. Ochell's Letter on Cholera .. .. .. .. .. .. .. .. .. .. .. .. .. 171
24. Rhinoplastic Operation .. .. .. .. .. .. .. .. .. .. .. .. .. .. .. 172
25. Mysterious Case .. .. .. .. .. .. .. .. .. .. .. .. .. .. .. .. .. 172
26. Delirium Tremens simulating Inebriety .. .. .. .. .. .. .. .. .. .. 173
27. Extra-uterine Pregnancy .. .. .. .. .. .. .. .. .. .. .. .. .. .. .. 173
28. On Torsion of the Arteries .. .. .. .. .. .. .. .. .. .. .. .. .. .. 174
29. Professor Delpech's Letter on Cholera .. .. .. .. .. .. .. .. .. .. 174
30. Permanent Contraction of Fingers, Operation for.. .. .. .. .. .. .. .. 175
31. Disease of the Heart .. .. .. .. .. .. .. .. .. .. .. .. .. .. .. .. 175
32. Caution to the Public—Hints on Scarlet Fever .. .. .. .. .. .. .. .. 176
33. Deficiency of the Cerebellum .. .. .. .. .. .. .. .. .. .. .. .. .. 177
34. Organization of Paganini .. .. .. .. .. .. .. .. .. .. .. .. .. .. .. 177
35. Pathology of Epilepsy .. .. .. .. .. .. .. .. .. .. .. .. .. .. .. 178
36. Remarkable Anomaly in the Blood-vessels .. .. .. .. .. .. .. .. .. 178
37. Retention of Urine .. .. .. .. .. .. .. .. .. .. .. .. .. .. .. .. 178
38. Inflammation of the Medullary Substance in long Bones .. .. .. .. .. 179
39. Memoir on Chlorotic Diseases.. .. .. .. .. .. .. .. .. .. .. .. .. .. 179
40. M. Velpeau's Historical and Critical Remarks on Hæmorrhage.. .. .. .. 181
41. New Medical Journal at Rio de Janeiro .. .. .. .. .. .. .. .. .. .. 182
42. Moreau de Jonnes on Population .. .. .. .. .. .. .. .. .. .. .. .. 182
43. Nutritious Soup from Bones .. .. .. .. .. .. .. .. .. .. .. .. .. .. 183
44. M. Esquirol on Contagion.. .. .. .. .. .. .. .. .. .. .. .. .. .. .. 183
45. On dilatation of the Cavities of the Heart .. .. .. .. .. .. .. .. .. 184
46. Varicose Tumour .. .. .. .. .. .. .. .. .. .. .. .. .. .. .. .. .. 184
47. Tumour of Spermatic Cord .. .. .. .. .. .. .. .. .. .. .. .. .. .. 185
48. Dissection after Death from Inanition .. .. .. .. .. .. .. .. .. .. 185

49. Roseola from Cubebs and Copaiba .. .. .. .. 185
50. Case of Lithotrity .. .. .. .. 186
51. M. Dance on a peculiar kind of Apoplexy .. .. .. .. 186
52. Chronic Hydrocephalus .. .. .. .. 187
53. Revulsive Bleeding in Affections of the Head .. .. .. .. 188
54. Cholera in Paris .. .. .. .. 189
55. Observations and Experiments on Respiration—(Original.) .. .. .. .. 190
56. Case of Tic Douloureux produced by Scirrhous Tumour .. .. .. .. 196
57. Mr. Rainey on Tests for detecting Arsenic .. .. .. .. 198
58. Drs. Vivenot on the Cholera of Vienna .. .. .. .. 199
59. Broussais's Lecture on Cholera .. .. .. .. 199
60. An Althelmintic Emulsion .. .. .. .. 201
61. Preternatural Lactation .. .. .. .. 201
62. Cases of Aneurism from Wounds .. .. .. .. 204
63. Case of Hepatic Abscess—Death .. .. .. .. 206
64. Dr. M'Cormac on Palm Oil .. .. .. .. 207
65. Secondary Hæmorrhage and successive Operation .. .. .. .. 209
66. Nitrate of Iron in Diarrhœa .. .. .. .. 214
67. (Review) Practical Treatise on Uterine Hæmorrhage in connexion with Pregnancy and Parturition By J. T. Ingleby.—*(First Article.)* .. .. .. .. 214
68. Analysis of the Blood and Urine in Diabetic Patients.. .. .. .. 219
69. Observations on certain Diseases of the Vascular System. By Dr. T. A. Wise, of the Bengal Medical Service—(Review).. .. .. .. 221
70. Cases of Abscess pressing on the Vagina. By Dr. Davis .. .. .. .. 222
71. Cases of Contraction of the Vagina. By Dr. Davis .. .. .. .. 224
72. Case of Abdominal Wound. By Mr. Hallett.. .. .. .. 226
73. Fatal Case of complete Cystocele. By Mr. Clement .. .. .. .. 228
74. Mr. Lizars on Cholera—(Review) .. .. .. .. 229
75. Aneurism by Anastomosis. Mr. Clement .. .. .. .. 232
76. Lithotomy in a Child. Mr. Clement .. .. .. .. 233
77. EXCERPTA CHOLERALOGICA.
1. Importation of Cholera—Irish Board of Health .. .. .. .. 234
2. The Parisian Outbreak—Professional Declaration .. .. .. .. 234
3. The Ely Board of Health—sagacious Deductions .. .. .. .. 237
4. Prognostications in Cholera .. .. .. .. 236
5. Baron Heurteloup on the Parisian Cholera .. .. .. .. 236
6. Modifications of Opinion—Lancet and Gazette .. .. .. .. 236
7. Cholera in Glasgow .. .. .. .. 238
8. Proofs of Identity—(Irish Proofs) .. .. .. .. 238
9. Fundamental Doctrine of Contagion—Mr. Orton's .. .. .. .. 239
10. Cholera in Cork—Proofs of travelling Cholera .. .. .. .. 239
11. Saline Injections in Cholera .. .. .. .. 240
78. Dr. Falrich's Wax Models.. .. .. .. 242
79. Mr. Marshall's Work on the Bills of Mortality .. .. .. .. 242
80. Cyclopædia of Practical Medicine—(Review.)
I. Auscultation—(Dr. Forbes) .. .. .. .. 246
II. Blood and Blood-letting—(Dr. M. Hall) .. .. .. .. 248
III. Bronchitis—(Dr. Williams) .. .. .. .. 250

81. Cases of Entero-vaginal Hernia. By Dr. Davis .. .. .. .. .. .. .. .. 253
82. Hackman on softening of the Spleen .. .. .. .. .. .. .. .. .. .. .. 257
83. Austrian Strictness in discharging Soldiers .. .. .. .. .. .. .. .. .. 260
Morality of young Recruits in Hot Countries .. .. .. .. .. .. .. .. 261
Malingering .. .. .. .. .. .. .. .. .. .. .. .. .. .. .. .. .. 262
84. Iodine for secondary Eruptions of Syphilis .. .. .. .. .. .. .. .. .. 263

Westminster Ophthalmic Hospital.

85. Ophthalmic Report from the above Institution .. .. .. .. .. .. .. .. 265
86. Cholera in the Ship Brutus .. .. .. .. .. .. .. .. .. .. .. .. .. 285
87. The late Dr. Dill.. .. .. .. .. .. .. .. .. .. .. .. .. .. .. .. 285
88. Medical Constitution .. .. .. .. .. .. .. .. .. .. .. .. .. .. .. 285
89. Dr. Seeds' Ophthalmic Lotion .. .. .. .. .. .. .. .. .. .. .. .. .. 286
Bibliographical Record .. .. .. .. .. .. .. .. .. .. .. .. .. .. .. 286

